#  Enhancing learning and retrieval of new information: a review of the forward testing effect

**DOI:** 10.1038/s41539-018-0024-y

**Published:** 2018-04-11

**Authors:** Chunliang Yang, Rosalind Potts, David R. Shanks

**Affiliations:** Division of Psychology and Language Sciences, University College London, London, WC1H 6BT UK

## Abstract

In recent years evidence has accumulated showing that interim testing of studied information facilitates learning and retrieval of new information—the *forward testing effect*. In the current article, we review the empirical evidence and putative mechanisms underlying this effect. The possible negative effects of administering interim tests and how these negative effects can be mitigated are discussed. We also propose some important directions for future research to explore. Finally, we summarize the practical implications for optimizing learning and teaching in educational settings.

Mastering a large body of knowledge or set of skills is a considerable challenge given the limits on our cognitive resources. Ever since the founding of experimental psychology, researchers have identified many efficient techniques to optimize learning and memory, such as structuring materials in a spaced way,^1^ administering quizzes or tests on learned information,^2^ creating concept maps,^3^ taking notes while learning,^4^ and so on.^5^ In the current article, we focus on a new technique recently developed for improving learning and retrieval of new information—administering interim tests during learning, which induces a beneficial *forward testing effect*.

Before introducing the forward testing effect, we briefly set the stage by describing the classic testing effect—the *backward testing effect*—to differentiate these two facilitatory effects of testing. The backward testing effect, which has been explored for over a century^6^ and in hundreds of studies,^5^ refers to the finding that testing of studied materials improves retention of those materials over restudying or doing nothing. For example, Roediger and Karpicke^2^ asked participants to study two passages, with one passage studied twice and the other studied once and tested once. In a test 1 week later, the tested passage was better recalled than the restudied one. The backward testing effect has been repeatedly demonstrated in numerous studies using different educational materials in both laboratories and classrooms (for reviews, see refs.^7,8^).

In addition, many studies have demonstrated that learning and testing of some information can boost the acquisition rate of new information.^9,10^ For example, Thune^9^ had participants study two lists of paired-associates across 2 days. On the first experimental day, participants studied a list of paired-associates, and then took a test on those pairs. Next, they restudied and were again tested on those pairs. This study–test cycle repeated until recall performance was perfect. On the second day, participants performed the same task all over again, except with a new list. Thune observed that participants required fewer cycles to reach the criterion on the second than the first experimental day. This facilitation effect is attributed to two psychological factors: “learning to learn” (i.e., prior learning and testing experiences teach people how to learn new information) and “warm-up” (i.e., prior learning and testing experiences warm people up and prepare them to master new information). Previous learning to learn and warm-up studies largely focused on how prior learning and testing experiences moderate subsequent learning of new information, and those studies did not include control conditions where learning took place without testing. Going beyond this, recent research has established that testing of studied information, by comparison with restudying or no treatment, can enhance learning and retrieval of new information—the forward testing effect.^11,12^

Although this forward effect has been identified fairly recently, many studies have accumulated exploring its robustness and limits. The most widely used experimental procedure for investigating the effect is illustrated schematically in Fig. 1. Two or three groups of participants are instructed to study a few blocks of computer-presented materials. Prior to study, all participants are warned that they will take a final, cumulative test on all to-be-studied materials. They are also informed that following the study of each block the computer will decide at random whether to give them an interim test on the material contained in that block. However, in fact, the interim test decisions are predetermined. In one experimental group, interim tests are administered after every block. In this review, we term this the Interim Test group. In one or two control groups, participants either take a distractor task (such as solving math problems) or restudy the material after each block except the final block, on which they are tested. We term these two control groups the Interim Distractor and Interim Restudy groups.

**Fig. 1:**
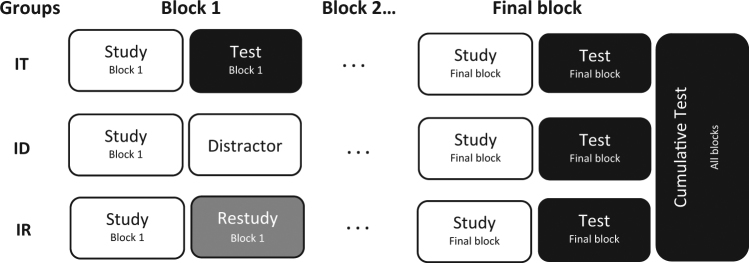
Experimental procedure for exploring the forward testing effect. The Interim Test (IT) group takes an interim test after studying each block. The Interim Distractor (ID) group completes a distractor task (e.g., solving math problems) after studying each block (except the final one) and takes an interim test on the final block. The Interim Restudy (IR) group restudies each just-studied block except the final one, and takes an interim test on the final block. All groups take a final, cumulative test following the interim test on the final block

Following the interim test on the final block, all three groups take a cumulative test on all blocks. The key finding is that the Interim Test group performs significantly better in the final block interim test than the control group(s). Almost all previous forward testing effect studies have observed the same result pattern in the cumulative test: the Interim Test group substantially outperformed the control group(s). For the sake of brevity, we do not discuss cumulative test results in this review. The critical finding in the research reviewed here is that despite all groups studying the same material in the final block, and taking an identical test on that material, learning and retrieval of that material is boosted if participants have previously been tested on preceding blocks. As we will describe, it has been established that this forward testing effect is a robust phenomenon across a variety of materials and contexts.

The present review aims to summarize the empirical findings on this topic, offer an overview of possible mechanisms underlying the effect, and discuss the practical implications for optimizing learning and teaching in educational settings. We also provide some suggestions for future research to further investigate aspects of this important effect that are currently poorly understood. The possible negative effects of interim testing on learning of new information and how to mitigate such negative effects are also discussed.

## Single item learning

Szpunar et al.^13^ conducted what is by now a classic study demonstrating the forward testing effect in single item learning. In their Experiment 3, they instructed three groups of participants to study five lists of words. An Interim Test group undertook an interim test at the end of every list, in which participants were asked to freely recall the words from the just-studied list. An Interim Restudy group restudied the previous list after studying each of Lists 1–4 and took an interim test on List 5. An Interim Distractor group solved math problems after studying each of Lists 1–4 and took an interim test on List 5.

Szpunar and colleagues found that the Interim Test group correctly recalled about twice as many List 5 words as the Interim Distractor and Interim Restudy groups in the List 5 interim test, which did not differ in their levels of recall. Meanwhile, the Interim Distractor and Interim Restudy groups committed about ten times as many intrusion errors from prior lists (proactive interference, PI; i.e., mistakenly recalling words from Lists 1–4) as the Interim Test group in the List 5 interim test. There was no significant difference in PI between the Interim Distractor and Interim Restudy groups. This study clearly reveals that interim testing of studied single items enhances learning and retrieval of new items compared to no interim testing (distractor task) or restudying. This finding has been repeatedly demonstrated in recent studies using word^12,14–20^ and picture lists.^21^

### Practical implications for instructors

Current research suggests that instructors can profitably administer low-stakes tests or quizzes to enhance students’ learning and memory of new single items and reduce the build-up of PI. For example, medical students need to master the names of the bones in the human skeleton. They may consecutively study the names of spine, chest, skull, arm, and leg bones. Medical teachers can administer interim tests following the teaching of each body part’s bone names to enhance students’ learning and retrieval of new names. In addition, interim tests on studied bone names will also prevent students from erroneously recalling other studied names (e.g., arm bones) when they are asked to recall new names (e.g., leg bones).

Nevertheless, we warn instructors to be cautious about the above proposal that interim testing reduces the build-up of PI because it is still unclear whether the effect of interim testing on release from PI will endure over the long term. To our knowledge, in all previous studies, the effect of interim testing on release from PI has been explored with a short retention interval (i.e., the interval between studying the final list and taking the interim test ranged from 0 to 5min). No study has yet investigated whether release from PI, induced by interim testing, is long lasting. This question awaits exploration in future research.

## Paired-associate learning

Following Szpunar et al.^13^ researchers began to explore the forward testing effect in paired-associate learning. For example, Weinstein et al.^22^ and Yang et al.^12,23^ asked two groups of participants to study four lists of face–name pairs. An Interim Test group took an interim test after studying each list whereas an Interim Distractor group only took an interim test on List 4. In the interim tests, all faces from the just-studied list were shown one-by-one and participants were asked to recall their corresponding names. The critical finding is that the Interim Test group correctly recalled about twice as many names as the Interim Distractor group in the List 4 interim test. All three studies also found that the Interim Distractor group experienced substantially more PI (i.e., mistakenly recalling names from Lists 1–3) than the Interim Test group. Moreover, the forward testing effect in paired-associate learning has also been established using foreign-translation word pairs such as Swahili–English^24^ and Euskara–English^12^ word pairs.

### Practical implications for instructors

Administering interim tests on studied paired-associates enhances learning and retrieval of new paired-associates and prevents the build-up of PI. Instructors can profitably administer low-stakes tests to promote students’ learning and retrieval of new paired-associate materials such as foreign-translation pairs, dates of historical events, simple definitions of scientific terms, and so on.

## Learning of complex materials

Researchers have also explored the forward testing effect in the learning of complex materials such as lecture videos and text passages. For example, Szpunar et al.^25^ instructed three (Interim Test/Interim Distractor/Interim Restudy) groups of participants to study an introductory statistics video, which was divided into four segments, each lasting approximately 5 min. Participants were allowed to take notes while watching the video, and they were asked to report whether their mind was “on task” (mind-wandering check) while watching the video. Szpunar and colleagues again obtained a forward testing effect in the Segment 4 interim test: The Interim Test group significantly outperformed the other two groups. They also found that the Interim Test group wrote down more notes and reported less mind-wandering than the other two groups.

Jing et al.^26^ conceptually replicated Szpunar et al.’s^25^ findings by employing a sociology lecture video as their experimental materials. Going beyond Szpunar et al.^25^ Jing and colleagues found that the Interim Test group reported more on-task mind-wandering (e.g., thoughts relating the lecture content to their own experiences) and less off-task mind-wandering (zoning out). On-task mind-wandering was positively related to later memory performance whereas off-task mind-wandering was inversely related to later recall. The forward testing effect in the learning of lecture video content has also been reported by Szpunar et al.^27^ and Yue et al.^28^. Yue et al. explored whether interim testing of a studied lecture video potentiates subsequent learning and retrieval of a new video. In their Experiment 2, Yue et al. had two (Interim Test/Interim Restudy) groups of participants study two scientific videos, with Video 1 concerning the life cycle of a star and Video 2 concerning lightning formation. In the Video 2 interim test, again, the Interim Test group outperformed the Interim Restudy group.

Wissman et al.^29^ explored the forward testing effect in the learning of prose passages. In their Experiment 1, they instructed participants to study a passage concerning the U.S. labor market, which was separated into three sections. An Interim Test group was tested after studying each section, whereas a No Interim Test group studied all three sections consecutively and was tested only on Section 3. In the interim tests, participants were asked to freely recall as much information as they could from the just-studied section. In the Section 3 interim test, the Interim Test group recalled about twice as much Section 3 information as the No Interim Test group. The forward testing effect in the learning of text passages has also been reported by Healy et al.^30^ and Zhou et al.^31^ using different text passages and test formats (e.g., multiple-choice tests).

Interim testing not only enhances memorization of specific content but also boosts information integration and comprehension of complex materials. For example, Jing et al.^26^ found that interim testing facilitates integration of related information within each segment and across different segments of a lecture video. Zhou et al.^31^ explored the forward testing effect in text comprehension. In a comprehension test, participants were required to combine several pieces of information to answer a given comprehension question. Zhou et al. observed that the Interim Test group substantially outperformed the Interim Restudy group, indicating that interim testing enhances text comprehension.

### Practical implications for instructors

Distance and online learning are becoming more and more popular^32^ and how to make these forms of learning maximally effective is a key concern for educators. As illustrated above, administering interim tests is an effective way to improve learning and retrieval of new information and reduce task-irrelevant mind-wandering. Therefore, instructors are encouraged to administer interim tests in online courses. Interim testing also benefits the learning (both memorization of specific content and comprehension) of text materials.

## Inductive learning

The aforementioned studies were largely restricted to exploring the forward testing effect in the learning of specific items (e.g., words, pictures, paired-associates, lecture videos, and passages). Yang and Shanks^33^ and Lee and Ahn^34^ explored the forward testing effect in inductive learning. For example, Lee and Ahn had three (Interim Test/Interim Restudy/Interim Distractor) groups of participants study 36 paintings, comprising six from each of six artists, in Block 1. Then the Interim Test group took a cued recall test on Block 1 paintings, in which the same 36 paintings were presented one-by-one in a random order and participants were asked to recall the corresponding artists’ names. The Interim Restudy group restudied the 36 paintings and the Interim Distractor group solved some math problems. In Block 2, all three groups studied 36 new paintings from another six artists. Next they took a classification test, in which 48 completely new paintings, comprising 4 from each of the 12 studied artists, were presented one-by one in a random order, with the 12 artists’ names presented simultaneously with each painting. Participants were instructed to select which was the correct artist for a given painting. The results showed that the Interim Test group substantially outperformed the Interim Restudy and Interim Distractor groups, revealing a forward testing effect in inductive learning (i.e., interim testing of studied concepts enhances learning and retention of new concepts; for similar findings, see ref. ^33^).

### Practical implications for instructors

Inductive learning is a key aspect of how humans learn and understand the world and a critical component of formal education. For example, fine art/history of art students are required to learn the painting styles of different artists; medical students are required to learn how to diagnose different diseases; students of linguistics have to learn the rules of a language; airport security screeners are required to learn how to detect threatening items by examining x-ray images. As illustrated by Yang and Shanks^33^ and Lee and Ahn^34^, interim testing during studying is an effective strategy for improving inductive learning.

## Self-regulated learning

All the aforementioned studies explored the forward testing effect in experimenter- or instructor-paced situations. But of course the pace of studying is often self-determined. Yang et al.^12^ investigated the forward testing effect in a self-paced situation. In their Experiment 1 they gave two (Interim Test/Interim Distractor) groups of participants as much time as they wanted to study five successive lists of Euskara–English word pairs. Again, Yang et al.^12^ obtained a forward testing effect in the List 5 interim test, with recall being higher in the Interim Test than in the Interim Distractor group. Interestingly, they also found that self-determined study time in the Interim Distractor group systematically decreased across lists whereas the decrease of study time was prevented by interim tests in the Interim Test group. When participants could choose how much time to devote to learning, their study time gradually dropped across successive lists in the absence of interim tests. However, when each list was followed by a test, participants maintained their list-by-list study time. This experiment, therefore, reveals that interim testing motivates people to commit more effort (study time) toward encoding new information in self-regulated learning, and shows that a simple intervention can motivate learners to devote more time to studying. This finding was conceptually replicated in Yang et al.’s^12^ Experiment 2, in which participants studied four lists of face–name pairs.

### Practical implications for instructors and learners

With the development of online courses and learning aids, self-regulated learning is becoming more and more common outside of the formal classroom.^35^ Yet we are far from being sophisticated learners^35^ and our self-regulated learning is often inadequate to achieve full mastery of the material we are studying.^36^ Administering interim tests during studying is a potent strategy to promote and sustain the effectiveness of self-regulated learning across a learning phase. Therefore, instructors can encourage learners to test themselves regularly when studying on their own, and learners are encouraged to administer interim tests during their own studying.

## Transfer of the forward testing effect

Students typically study different types of information from class to class. A biology class may be followed by a history class, for instance. It is therefore important to explore the transferability of the forward testing effect—whether interim testing of studied information in one domain can enhance learning and retrieval of new information in a different domain.

Yang et al.^23^ conducted three experiments to explore this question. In their Experiment 3, two (Interim Test/Interim Restudy) groups of participants were asked to consecutively study three blocks of factual statements followed by a block of paintings. In each of Blocks 1–3, both groups studied 10 statements concerning artists’ contributions to art (e.g., *Veronese introduced a greater realism and sumptuous, decorative color*). The Interim Test group took a fill-in-the-blank test (e.g., *Veronese introduced a greater _____ and sumptuous, decorative color*) following studying each block, whereas the Interim Restudy group restudied the statements. In Block 4, both groups studied 48 paintings, comprising 6 from each of eight new artists, and then took a classification test in multiple-choice format. In the classification test, 32 new paintings, comprising 4 from each of the eight artists, were presented one-by-one, in a random order, with the eight artists’ names presented below each painting, and participants were required to identify who the correct artist was for each painting. The results showed that the Interim Test group classified more paintings correctly than the Interim Restudy group, revealing transfer of the forward testing effect from low-level (verbal text) to high-level (visual concept) learning. Yang et al.’s^23^ Experiments 1 and 2 also obtained transfer of the forward testing effect using different educational materials.

Because the study described above did not include groups which studied materials from the same domain in each block, it is unknown whether the forward testing effect is weaker with a change in domain compared to no change in domain. Hence, to what extent the standard forward testing effect is attenuated by a change of material or by a change of test format is currently unknown and must await further research.

### Practical implications for instructors

The forward testing effect transfers among different domains of learning, even when test formats are switched across a study phase. Instructors can safely administer interim tests during lectures to enhance learning of new information even when different topics are covered and different types of tests are administered during the course of a lecture. For example, in a class, medical students may study some scientific definitions and then move on to learn diagnostic techniques, or statistics students may study properties of distributions and then learn the steps involved in conducting a test in a software package. Tests administered after the first of these learning episodes are expected to enhance learning and retrieval of the subsequent information.

## Underlying mechanisms

Mechanisms that operate during either the encoding or retrieval phase, or both, may contribute to this facilitatory forward effect of interim testing and many possible explanations have been proposed to account for this effect. Here we briefly review these explanations. It is important to emphasize at the outset that these accounts are not mutually exclusive, that most are at a preliminary stage of development, and that few have been subject to direct testing of their key predictions. To aid understanding, we classify the accounts along two major dimensions, whether they regard encoding or retrieval as the main locus of the forward testing effect, and whether or not they propose that the effect is mediated by changes in motivation (see Table 1).

**Table 1:** Theories proposed to account for the forward testing effect

Theories	References	Descriptions	Motivational?	Active phases
Release from PI	Szpunar et al.^13^	Interim testing induces context changes between blocks, which reduce the build-up of PI and facilitate recall of target (new) items	No	Retrieval
Encoding reset	Pastötter et al.^15^	Interim testing induces context changes between blocks, which “reset” subsequent encoding of new information and make it as effective as the encoding of prior information	No	Encoding
Activation facilitation	Wissman et al.^29^	Interim testing induces greater retention of tested information and makes the tested information more active while encoding new information, which helps encoding and comprehension of new information	No	Encoding
Encoding strategy	Cho et al.^24^	Interim testing induces more effective encoding strategies than no interim testing	No	Encoding
Retrieval strategy	Cho et al.^24^	More effective retrieval strategies are developed during prior interim tests, which facilitate recall of target (new) items in the subsequent interim test	No	Retrieval
Test expectancy	Weinstein et al.^14^	Interim testing induces a greater expectancy of an immediate interim test, which motivates more effort toward encoding new information	Yes	Encoding
Failure-encoding-effort	Cho et al.^24^	Retrieval failures in prior interim tests induce dissatisfaction and motivate more effort toward encoding new information	Yes	Encoding
Retrieval effort	Cho et al.^24^	Retrieval failures in prior interim tests motivate more effort to retrieve the target (new) items in the subsequent interim test	Yes	Retrieval

It has been well-established that testing during learning can induce context changes.^37^ Szpunar et al.^13^ postulated that the forward testing effect is mainly caused by the fact that context changes, induced by interim tests, reduce the build-up of PI and improve the recall of new information—we term this explanation the *release from PI* theory. Interim testing of studied items updates these items’ mental contexts, and hence these studied/tested items are associated with both a study (S) and a retrieval (R) context.^37^ Following the interim test on studied items, participants then study some new items, which are only associated with a study (S) context. In the subsequent interim test in which they are required to recall the target (new) items, the context difference between the previous items, which have been both studied and tested (and associated with both contexts S and R), and the new items (only associated with context S) facilitates differentiation between these items and reduces the impairment from PI. The above-reviewed studies, which explored the forward testing effects in single item learning and paired-associate learning, offer strong support for the release from PI theory: These studies observed that interim testing of studied items reduces the build-up of PI.

Different from the release from PI theory, which focuses on the influences of context changes on subsequent recall of new information, an *encoding reset* theory, proposed by Pastötter et al.,^15^ focuses on the influence of context changes on subsequent encoding of new information. Specifically, this theory postulates that interim tests induce context changes between blocks, which in turn induce a “reset” of subsequent encoding, making it as effective as the encoding of material in prior blocks. Indeed, Pastötter et al.^38^ found that an imagination task (e.g., participants imagined walking through their parents’ living room), which induces mental context changes between the studying of two lists of words, makes the learning of the second list as effective as that of the first list. In contrast, in the absence of the imagination task, less attention is attached to the encoding of the second list compared to the encoding of the first one.

Both the release from PI and encoding reset theories focus on the roles of context changes in the forward testing effect. Nonetheless, both theories have difficulty explaining the forward testing effect observed in an important study by Wissman et al.^29^ In their Experiment 4, Wissman and colleagues had three groups of participants study a three-section passage. An Interim Test group took a free recall test after studying each section, an Interim Distractor group solved math problems after studying each of Sections 1 and 2 but took a free recall test on Section 3, and a Section-3 group only studied Section 3 and took a free recall test on it. The results showed that the Interim Test group recalled about twice as much information from Section 3 as the Interim Distractor and Section-3 groups. This is striking because according to the release from PI theory, recall in the Section-3 group should be better than or at least equal to that in the Interim Test group, for whom at least some PI would accumulate across the study phase. Similarly, according to the encoding reset theory, recall in the Section-3 group should be better or at least equal to that in the Interim Test group, because the Section-3 group’s encoding of Section 3 material should be at least as effective as that of the Interim Test group. However, recall in the Section-3 group was, in fact, poorer than that in the Interim Test group.

Hence, Wissman et al. proposed an *activation facilitation* theory to account for their forward testing effect. This theory postulates that greater activation and retention of learned information, induced by prior interim tests, can facilitate encoding of new related information, especially for complex materials such as lecture videos and text passages. Different sections of a passage or a lecture video are related. Interim testing of prior sections improves retention of tested information compared with restudying or doing nothing. While encoding the target section (new information), the tested information is more activated and accessible on this theory, which in turn facilitates comprehension of new information.^39^

Besides the three theories discussed above, Cho et al.^24^ proposed that the forward testing effect may be produced by encoding strategy changes—the *encoding strategy* theory. This theory hypothesizes that prior interim tests inform people what kind of test to expect and accordingly they adjust their encoding strategies, which facilities subsequent encoding of new information. Previous studies have shown that testing can foster the development and adoption of more effective learning strategies.^40–42^ For example, Soderstrom and Bjork^42^ found that, following testing, individuals are likely to employ more effective encoding strategies (e.g., relating the item to something that is meaningful to them) than they are following restudying. Cho et al.^24^ also proposed a complementary *retrieval strategy* theory to account for the forward testing effect, which postulates that prior interim tests on studied information help people to adopt more efficient retrieval strategies. Specifically, this theory postulates that participants gradually develop more effective retrieval strategies across successive interim tests (for an illustration that prior interim tests induce retrieval strategy changes, see ref. ^43^), and these more effective retrieval strategies facilitate recall of new items in the subsequent interim test.

We have summarized five (release from PI; encoding reset; activation facilitation; encoding strategy; retrieval strategy) theories explaining the forward testing effect. Nonetheless, Yang et al.^23^ proposed that all these five theories have difficulty explaining the transfer of the forward testing effect obtained in their Experiment 3. First, a switch of material types led to no PI in the Block 4 interim test, and materials in Blocks 1–3 (text statements) and Block 4 (paintings) were from different domains and completely unrelated. Hence, transfer could be accounted for by neither release from PI nor activation facilitation. Second, a switch of material types also induces substantial context changes between blocks, which should “reset” subsequent encoding regardless of the inclusion or not of a test.^18,23,44,45^ Therefore, the encoding reset theory is also unlikely to account for successful transfer. Third, given that material types and test formats (fill-in-the-blank tests in Blocks 1–3 and a multiple-choice test in Block 4) were changed, there is little reason to expect that participants could have developed and adopted more effective encoding/retrieval strategies across Blocks 1–3 that would be applicable in Block 4. Hence, the encoding/retrieval strategy theories also have difficulty explaining the transfer. Yang et al.,^23^ therefore, suggested that the transferability of the forward testing effect that they observed in their Experiment 3 was best explained by enhanced motivation (that is, prior interim tests motivate people to exert more effort toward encoding/retrieval of new information).

Three specific possibilities have been put forward to explain why interim tests might enhance people’s motivation toward encoding/retrieval of new information. Weinstein et al.^14^ suggested that it is caused or mediated by test expectancy. We term this explanation the *test expectancy* theory. This theory postulates that, since the Interim Test group is always tested on prior blocks, they should have a high expectancy of an interim test on the next list, and high test expectancy motivates people to exert greater effort toward encoding new information.^14,23,46^ To test this idea, Weinstein et al. asked an Interim Test and an Interim Distractor group to study five lists of words. Before studying each list, all participants were instructed to report how likely they thought it was that they would be asked to take an immediate interim test on the next list. The results showed that the Interim Test group’s test expectancy increased whereas the Interim Distractor group’s decreased across lists. Yang et al.^23^ observed the same result pattern in their Experiments 1–3.

An alternative explanation for why interim tests might motivate individuals to devote greater encoding effort was proposed by Cho et al.,^24^ who postulated that it is retrieval failures in prior interim tests that motivate them to commit more encoding effort. We term this theory the *failure-encoding-effort* theory. Retrieval failures in prior interim tests induce dissatisfaction about prior learning as well as awareness of the difficulty of achieving successful recall, leading to enhanced study effort to mitigate this dissatisfaction. Consistent with this idea, previous studies have shown that retrieval failures or committing errors in prior tests can potentiate subsequent encoding.^47–49^

Finally, besides enhanced encoding effort (induced by enhanced test expectancy and/or retrieval failures in prior interim tests), enhanced retrieval effort may also play a role in the forward testing effect.^24^ For instance, Cho et al.^24^ attributed the forward benefit of interim testing to enhanced retrieval effort—the *retrieval effort* theory: Retrieval failures in prior interim tests might induce dissatisfaction about prior interim test performance and then motivate participants to exert more retrieval effort in subsequent interim tests to alleviate their dissatisfaction. Evidence supporting the retrieval effort theory comes from Yang et al.’s^23^ Experiment 3. In the Block 4 interim test, Yang et al. assessed participants’ retrieval effort by measuring how much time they spent classifying the paintings. The retrieval effort theory predicts that the Interim Test group would spend more time (an index of retrieval effort) classifying the paintings than the Interim Restudy group. Yang et al.’s Experiment 3 affirmed this prediction.

Overall, we have discussed at least eight possible theories, each proposing a mechanism that may underlie the forward testing effect. Some theories are similar. For example, the retrieval effort theory can be regarded as a subset of the retrieval strategy theory, as committing more effort during retrieval is a form of retrieval strategy change. Similarly, committing more effort during encoding can be seen as a form of encoding strategy change. Cho et al.^24^ noted that enhanced effort is a quantitative change whereas encoding or retrieval strategy change is a qualitative change. Quantitative changes refer to the changes in the magnitude of effort people devote to a task, whereas qualitative changes mainly refer to alterations in the ways people encode and retrieve items.

These eight theories can be divided into two clusters according to the phase at which the putative mechanism they describe is assumed to be active: encoding (encoding reset; activation facilitation; encoding strategy; test expectancy; failure-encoding-effort) and retrieval (release from PI; retrieval strategy; retrieval effort). They can also be divided according to their proposal about the role of motivation: motivational (test expectancy; failure-encoding-effort; retrieval effort) and non-motivational (release from PI; encoding reset; activation facilitation; encoding strategy; retrieval strategy). Although we divide the mechanisms these theories propose into different clusters, we reiterate that they need not be mutually exclusive and some of them may operate in parallel in some situations and produce overlapping forward testing effects.

For different materials and in different situations, some mechanisms may play the main roles and others may play lesser or even no role. For example, for complex materials (e.g., passages, lecture videos), there may be no PI and therefore the release from PI mechanism may play little or even no role.^29^ In contrast, the activation facilitation mechanism may play an important role in the learning of complex materials.^29^ For single items (e.g., unrelated word lists), activation, and retention of prior information cannot aid comprehension of new information and therefore the activation facilitation mechanism may play a small or even no role, while the release from PI mechanism may play a more important role.^12,13^ Future studies are needed to further explore these possible mechanisms and investigate in which situations and for which materials the different mechanism(s) contribute to the forward testing effect.

## Individual differences

In the studies reviewed above, participants were mostly college students. Can we generalize our conclusions about the forward benefits of testing to other groups? There is some evidence that the effect occurs in a range of participant groups, although further research is needed. Pastötter et al.^21^ explored whether the forward testing effect generalizes to individuals who have suffered traumatic brain injury (TBI). TBI is associated with many memory deficits. For example, it affects short-term much more than long-term memory.^50^ For individuals with TBI, their memory of past events (e.g., their childhood memory) is relatively intact but they suffer deficits in remembering recent events. Pastötter et al.^21^ asked TBI and healthy individuals to study three lists of line drawings of common objects. In the List 3 interim test, Pastötter et al.^21^ obtained a forward testing effect in both TBI and healthy individuals, indicating that interim testing during learning can be used to reduce memory deficits in people with TBI. As yet, it is still unknown whether interim testing can be used to mitigate memory deficits caused by other diseases such as Alzheimer’s disease, ADHD, and multiple sclerosis. Future research could usefully investigate this.

Aslan and Bäuml^17^ explored the forward testing effect in children. They asked adults, older (average age = 8.8 years), and younger children (average age = 6.7 years) to study four lists of words. Aslan and Bäuml obtained a forward testing effect in adults and older children but not in younger children. They observed that, for older children and adults, the interim test groups suffered from less PI than the Interim Restudy groups in the List 4 interim test, whereas for the younger children there was no difference in PI between the Interim Test and Interim Restudy groups. Aslan and Bäuml speculated that the absence of the forward testing effect in younger children’s single item learning may result from their deficits in inhibition of PI, because for younger children the interim tests did not reduce the accumulation of PI across lists.

The absence of the forward testing effect for younger children in single item learning does not necessitate the absence of this effect in the learning of complex materials, as the activation facilitation and enhanced encoding effort mechanisms may play important roles for complex materials whereas the release from PI mechanism is likely to play little role.^29^ Future research could profitably explore this issue.

## Possible negative effects of interim testing

We have summarized the facilitatory effects of interim testing in the learning and retrieval of new information. However, recent research has shown that in some situations interim testing can lead to negative effects. Interim testing motivates people to exert more effort to the encoding of new materials. It has been suggested that, when the tested and new materials are presented together, the tested materials may “forcibly occupy” the encoding time and borrow the limited time available for studying new materials––the *borrowed time effect*.^51^

As an illustration of this, Finn and Roediger^52^ asked two (Interim Test/Interim Restudy) groups of participants to study some face–name–profession associations. In the first encoding phase, both groups studied face–name pairs one-by-one, for 5 s each. Following a short distractor task, the Interim Restudy group restudied all face–name pairs one-by-one, for 5 s each, and immediately following the presentation of each face–name pair, the same face with its name and profession were presented simultaneously for 5 s for participants to study. In contrast, following the distractor task, the Interim Test group took an interim test, in which they were asked to recall each face’s corresponding name, and immediately following this recall, the same face was shown with its corresponding name as corrective feedback for 2 s. After that, the face with its name and profession were simultaneously presented on screen for 5 s. Twenty-four hours later, both groups took a final test, in which participants were shown the faces one at a time and asked to recall each face’s corresponding name and profession.

The results showed that the Interim Test group correctly recalled more names than the Interim Restudy group, whereas the recall of professions showed the reverse pattern: The Interim Restudy group recalled more professions than the Interim Test group. In recent follow-up research, Davis and Chan^51^ proposed that the interim test on face–name pairs led the Interim Test group to continue to focus on learning the names when they were shown the face–name–profession pairs, as the prior interim test made them aware of the difficulty of remembering the face–name pairs. This focus on the learning of names “borrowed” encoding time and resources which would otherwise be spent on learning professions.

Davis and Chan^51^ showed that this negative effect can be completely reversed. In their Experiment 4, they separated the interim test on face–name pairs and the encoding of face–profession pairs. Following initial study of the face–name pairs, an Interim Restudy group restudied all face–name pairs one-by-one, whereas an Interim Test group was shown the faces one-by-one and was asked to recall their names, and corrective feedback was given in the interim test. Then both groups were asked to study the face–profession pairs, and in this phase, no names were shown alongside. At the end of this study phase, participants took a final test in which they were asked to recall the names and professions in response to the faces. The results showed that, in the final test, the Interim Test group recalled more professions than the Interim Restudy group. Hence the Davis and Chan^51^ study showed that separate presentation of tested and new information can not only eliminate the borrowed time effect (the finding that interim testing on face–name pairs impairs the learning of face–profession pairs when face–name–profession information was presented simultaneously) but can also induce a positive forward testing effect (interim testing on face–name pairs enhances the learning of face–profession pairs when the associations are separated). Unfortunately, the positive testing effect for the face–name pairs was also reversed: now testing impaired memory for the names relative to restudy. In a more recent study, Davis et al.^53^ provided evidence suggesting that the impairment to new learning could be due to a task switching cost rather than to time borrowing, though the two accounts are not mutually exclusive.

Finn^54^ reported finding that, contrary to the predictions of the borrowed time hypothesis, giving participants unlimited time for test and review of feedback, thereby minimizing the need to borrow time, failed to eliminate retrieval-impaired learning of new information. While time borrowing may account for some of the data, it is therefore unlikely to be a complete explanation for retrieval-impaired learning of complementary associations (for other possible explanations about why interim testing may impair learning of new information, see refs.^52–54^). Finn and colleagues’ finding (that interim testing impairs the learning of new complementary information when tested and new information is simultaneously presented) is intriguing and research into this phenomenon is at an early stage. The need for further research concerning this question is pressing.

### Practical implications for instructors

Clearly, there will be many occasions in the classroom when an instructor asks students to recall a piece of studied information (e.g., A-B associates) as a prelude to introducing new, complementary information (e.g., A-C associates). Finn and colleagues’ studies^52,54^ suggest that simultaneous presentation of tested and new information (e.g., A-B-C associations) following retrieval of studied information (e.g., A-B associates) can impair the learning of new information (e.g., A-C associates) but the mechanisms underlying this effect are not yet clearly delineated. The finding that impairment of new learning is greatest in situations characterized by frequent task switching suggests that it may not pose a serious problem in classroom situations, where such frequent switching is unlikely, but not enough is yet known about the boundary conditions of the effect to make firm recommendations regarding classroom instruction.

## Future research directions

Table 2 depicts some directions for future research to explore. As discussed above, more work is needed to investigate the mechanisms underlying the forward testing effect and individual differences in the magnitude of this effect. For example, older adults, like younger children, have difficulty in inhibiting PI. Does the forward testing effect generalize to older adults? To date, little neuroscientific research has been conducted to explore the human brain networks involved in the forward testing effect.^55^ Future neuroscience research is needed to begin to fill this gap. The backward testing effect has been repeatedly demonstrated in real classrooms but the forward testing effect has not been yet. Of high priority for future research is to test whether the forward effect generalizes to classroom settings. To date, forward testing effect studies have focused on the short-term outcomes of the effect: the target (final) block’s interim test was administered immediately following its learning phase. Future research could profitably explore the long-term outcomes of the effect.

**Table 2:** Future research directions for investigating the forward testing effect

Suggested future research directions
1. Further investigation of the possible mechanisms underlying the forward testing effect, and testing of key predictions made by different theoretical accounts.
2. Does the forward testing effect generalize to individuals with Alzheimer’s disease or ADHD?
3. Can young children’s learning of complex materials benefit from interim testing?
4. Does the forward testing effect generalize to older adults?
5. What brain networks are involved in the forward testing effect?
6. Testing the forward testing effect in the classroom.
7. What are the long-term outcomes of the forward testing effect?

## Summary

Learners and educators sometimes simply regard testing as a tool for assessing one’s learning status. Some educators even propose to minimize testing in the classroom as they think it is time-consuming^7^ and scoring of tests excessively demanding. However, numerous previous studies have confirmed the reliability of the backward testing effect in laboratories and real classrooms, even with low-stakes quizzes.^7^ Furthermore, the above-reviewed studies have demonstrated the reliability of the forward testing effect across a variety of educational materials. Therefore, the forward and backward testing effects, jointly, make a strong case for learners and instructors to administer interim tests or quizzes during learning.

Interim testing can not only enhance learning and retrieval of new information but also prevent the build-up of PI.^12,13,22^ In real-world learning settings, students frequently suffer from PI. For example, in a geography class, students may need to master the basic information (e.g., geography, culture, economy, demographics) of a few European countries (e.g., Norway, Denmark, and Spain). Students may confuse information relating to different countries. Therefore, understanding how to prevent the build-up of PI is critical for instructors and learners in such situations. As Yang et al.^12^ showed, interim testing significantly decreases the build-up of PI regardless of whether learning is self- or instructor-paced. Therefore, instructors and learners are encouraged to administer interim tests to prevent the build-up of PI. We again warn instructors to be cautious about this proposal, however, because it is unknown whether release from PI, induced by interim testing, is long lasting.

To summarize, interim testing is a powerful technique in optimizing learning of new information. Studies using a variety of educational materials have shown that the forward testing effect is a robust phenomenon. Interim testing can be used to enhance the learning of new single items, paired-associates, complex materials, and concepts (categories). It not only benefits memorization of specific content but also boosts information integration, producing superior knowledge organization. The forward testing effect is not limited to instructor-paced situations but also generalizes to self-paced ones; it is not limited to healthy individuals but also generalizes to individuals with brain injury; it is not limited to the same type of material but is also transferable to different types of material (and different test formats); it not only enhances learning and retrieval of new information but also prevents the build-up of PI. Both variations in the encoding and retrieval phases may contribute to the forward testing effect. Although interim testing may impair learning of new information when tested and new materials are presented together, this negative effect can be eliminated and reversed by the separate presentation of tested and new information. Further investigations on aspects of this important effect, which are currently poorly understood, are needed.
